# *‘I decided to attend to him because it’s my duty’*: Student Nurses perception and attitude towards care of older adults

**DOI:** 10.1186/s12909-021-03090-z

**Published:** 2022-01-08

**Authors:** Priscilla Yeye Adumoah Attafuah, Ninon Amertil, Jacob Owusu Sarfo, David Atsu Deegbe, Delight Nyonator, Caleb Amponsah-Boama, Aaron A. Abuosi

**Affiliations:** 1 School of Nursing and Midwifery, University of Ghana, Legon, Ghana; 2 Department of Health Services Research and Care and Public Health Research Institute (CAPHRI), Maastricht University, Maastricht, the Netherlands; 3 School of Nursing and Midwifery, Valley View University, Oyibi, Ghana; 4 Department of Health, Physical Education and Recreation, University of Cape Coast, Cape Coast, Ghana; 5 Department of Public Admin and Health Services, University of Ghana Business School, Legon, Ghana

**Keywords:** Career choice, Older adults, Nurse's attitude, Student perception

## Abstract

**Background and Aim:**

Nurses perception and attitude towards an older patient can positively or negatively influence the quality rendered. As students under training, the views of this population needs to be sought and shaped to improve the quality of care the older patients receive. This is because life expectancy is on the rise. The study aimed to explore students’ perception of ageing and their attitude towards care of the older adults.

**Methods and materials:**

An exploratory descriptive design was used. Data form containing the sociodemographic attributes of the students and a semi-structured interview form developed by the researchers in line with the literature. The participants interviewed were student nurses who had been in clinical practice for at least one semester. Four focus group discussions (FGD) were held.

**Results:**

Average age of the participants was 22.30 years. An equal number of males and females (15 each) were recruited to have a balance in gender. Students expressed that they saw the older adults as their grandparents so they try to accord them respect and care. However, older adults are perceived not receptive to nurses in training. The students stated that registered nurses neglected the basic care of older adults such as diaper changes, bathing, and feeding, and would rather beckon student nurses to attend to the older adults.

**Conclusion:**

Gerontology as a stand-alone course is necessary for early years of training to give an in-depth education to nursing students and instil a positive attitude towards older adult patients.

## Introduction

Nursing education and practice complement each other to improve care rendered to patients. Student nurses receive training to take care of the sick in various practice settings. Geriatric care is one of these settings. People worldwide are managing their health to live longer. Globally, the population of older people is increasing daily because of improved technology and diverse ways of sharing information [4, 14, 28, 29]. The UN (2019) defined older persons as people 60 years and over. Additionally, the age range for defining an older adult in Ghana is 60 years because that is the stipulated age for retirement. Nonetheless, increasing age comes with an increased probability of various health conditions because of the degeneration of body systems. With the increased life expectancy [14, 29], the care of older adults is prone to be a challenge [26] in the future if steps are not taken to put solutions in place. As stipulated by the WHO, ageism is a global issue that needs to be tackled [25]. Ageism, a complex phenomenon of discrimination based on age, involves both positive and negative perceptions of older adults [5]. The quality of care of the older adult can be influenced by ageist attitudes held by nurses providing this care. Speaking rudely or disrespectfully to an older adult and/or rendering fragmented care to them are examples of ageist behaviours [7].

Currently, care of the older adult patients in Ghana is not in separate units like the paediatrics unit. They are seen together with the adults and youth in the general wards. Studies have shown that nurses with specialist training are better equipped to render care to patients [19, 20]. Looking at the curriculum of most nursing schools, gerontology is usually not a stand-alone course [12, 15, 29] but students are expected to learn it in their medical-surgical nursing classes. Hence, training or education of student nurses regarding the care of older adults is an area that is not taught in-depth [9, 17, 27, 31]. With the rising number of geriatrics [6, 11, 13, 29], the issue of ageism is still under-researched in sub-Saharan Africa. In nursing, it is important to address the issue of hostile and benevolent ageism as it is expected that nurses will be given the requisite skills to care for all older adults [12, 26].

In Africa, it is theorized that older adults are respected because of the culture of society. Titles of especially the older adults are very necessary and not calling an individual with the appropriate title is perceived as a sign of disrespect. The attitude of student nurses towards caring for older adults needs to be examined and shaped, to meet the ideals of culture [12], 18]. Studies, which showed negative attitudes of student nurses towards the care of older adults, have been carried out in various countries [8], while others also documented positive attitudes [16, 18, 26]. A study by Rathnayake, et al., [29], showed an almost 50-50 (49-44) ratio for those with positive attitudes and those with negative attitudes.

Faronbi, Adebowale, Faronbi, Musa, & Ayamolowo, ([6]) reported a positive attitude among student nurses towards older adults in a study in Nigeria. They however recommended a qualitative study to explore the perception and attitudes of these students. It is reported that students over the years prefer to work in general adult wards and paediatric wards at the expense of the older adults [12, 15, 29]. Hostile ageism deters the care of older adults and the desire to specialize in gerontological nursing among current and future nurses [7]. It is disturbing that the older adult population is growing but interest in the attitude of health personal towards this group is not rising simultaneously. The quality of nursing care older adults will receive is highly reliant on the training of today’s student nurses. Student nurses have had contact with older persons and it is necessary to assess their perception of older adults care as well as their attitude towards care of the older adults. The authors also sought to identify any students with an interest in career progression in the field of geriatrics.

## Method

### Study design

An exploratory descriptive design was used. This enables researchers to explain a phenomenon from the perspective of the individuals being studied (student nurses) in their natural setting (the university).

### Study Procedure, Participants and Recruitment

This is a qualitative study using an exploratory descriptive design, conducted among nursing students, who have had exposure to the clinical setting. The study was carried out at a private university in Ghana. Details are blinded for review. A semi-structured interview guide was developed using questions from Kogans’ Attitudes toward Old People scale [16] to guide the focus of the research. Data forms containing the sociodemographic attributes of the students and a semi-structured interview guide were developed by the researchers in line with the literature. Eligibility was for all students who had experienced at least a semester of clinical attachment. Class leaders recruited participants, and those who volunteered were given appointments by the first author per available slots.

Participants were informed that they will be recorded however, they do not need to use real names for anonymity. Verbal consent was given and they were permitted to withdraw if they so wish. Four focus group interviews were conducted in this study. Each group was made up of five participants. Although this group size has been criticised by Merton, Fiske and Kendall (1990) as small, Greenbaum (1988) justifies this number as a mini-group. Nonetheless, Kitzinger (1996) and Twinn (1998) suggest four to eight and four to five respectively is an ideal size as it allows for increased interaction among group members.

Each interview lasted for 30-45 min. Focus group discussions were held in an appropriate room in the university. Before beginning the focus group discussions, participants gathered in a room and were seated around a table facing the interviewer, and the aim of the study was explained to them. Participants were assigned a pseudonym to be used instead of their actual names and all discussions were tape-recorded. Furthermore, trustworthiness in the study was ensured by following Guba and Lincoln’s (1989) four criteria (credibility, dependability, confirmability, and transferability) to ensure qualitative rigour.

### Ethical Considerations

 Written approval was obtained from the School of Nursing following institutional ethical clearance from Dodowa Health Review Centre. The purpose of the study and the rights of participants were announced to all the nursing students. Informed consent was obtained from interested students after the objectives, method of the research, and their rights were explained.

### Analysis

Content analysis data was carried out alongside data collection. Verbatim transcription of interviews was done and read severally to precisely comprehend the views of student nurses by two independent coders. First, codes were allocated to meaningful portions after getting the core of the data. Similar codes were carefully assembled to form subthemes and subsequently, themes. Differences in coding or cataloguing were discussed by the two researchers until agreement was reached. The researchers deliberated on the themes generated and ensured that the data were free of personal biases. The themes were then put under the various objectives set for the study; “perception of older adults”, “attitudes towards older adults” and “interest in career progression as a geriatric nurse”. The data was finally exported to NVivo version 11 which was used to manage the data. Thick verbatim quotations were also used to support the findings of the study which revealed the perception and attitudes of student nurses when caring for older adults.

## Results

### Socio-Demographic Characteristics

The average age of the participants was 22.30 years. An equal number (15) of males and females were recruited to have a balance in gender. They were all undergraduate students who have had at least one semester of clinical interaction. There were five each from level 100 and 200 and twenty from level 400, which is the final year. Level 300 students were not on campus during the time of the interview.

Three main themes and six subthemes that emerged from the analysed data of this study are presented in Table 1.

**Table 1:** Themes, subthemes, and codes

Themes	Subthemes	Sample codes
Perception	Perception of ageing concept Perception of problems of older adult patients	chronological age, physical appearance, activity level excessive complaints, communication issues, grieving, depression, loneliness, domineering, overly dependent, forgetfulness, poor gait
Attitude	Attitude towards care of older adults Perception of the attitude of registered nurses	Grandparents, Opportunity to learn, Patients, Duty. Kind, patient, Humble, rude, neglect
Career progression	Career progression into geriatric nursing needs Desire to progress into geriatric nursing	Knowledge and Skills Patience Maybe, Not all, Definitely,

### Perception

This theme describes the students’ opinions about the concept of ageing and the perceived problems of older adults. Participants narrated their views on who they referred to as an older adult and some problems they feel older adults experienced. An individuals’ physique and age contribute to influencing how student nurses identify an older adult.

#### Perception of ageing concept

All participants expressed their views on the concept of ageing. Most participants used the chronological age to describe an older person. A few participants expressed the physical appearance determined if one was old. Additionally, only two participants identified an individual as an older adult based on the activity level.

“*I think it is dependent on how active the person is. Someone can be 70 years and still be active more than someone who is 55*” **FGD3, K3**.

#### Perception of problems of older adults

Participants indicated that older persons had problems with students attending to them. A few participants stated that older adults welcomed their care. However, more than half of the participants experienced rejection from older adult patients. Two students shared their experience as follows:

*“They didn’t understand why a young person should be taking care of them. Often made statements like: a small girl like you can you nurse me?”* **FGD2**, ***A2****“When I was about nursing him, he said he would like the staff nurses to nurse him. With no reason”* **FGD3, AK**.

All articipants identified communication as a problem for older adults though in various aspects. Some older adults were seen to be talkative and good at making conversation but a few kept to themselves and ignored any gestures from students.

*“… Either they did not understand the expressions of the student, or they talked too much or just would not talk at all (*not because they were unable to but just didn’t want to*). And the woman was very interesting. She was good at conversation.* **FGD3, K3**.

More than half of the participants had encountered older persons who were always depressed, sad or lonely. these participants all narrated that the older adults with these characteristics were usually very old and at the end of life.

*“79 years old, he didn’t understand why we were bothering ourselves to nurse him. …felt we should leave him to die and didn’t want to cooperate with any procedure”* **FGD2, KW2**.

*“I nursed a patient who was always sad. They start their grieving very early”* **FGD4, N**.

Some of the students also stated that, they observed that some older persons struggle with shyness.

*“It is difficult for them to cooperate when we are attending to them. … They are either shy or don’t think we are capable”* **FGD3, KA**.

However, a few participants observed some expressions of domination and overly dependence on the part of older adult patients.

*“the patient I had recently, was a bossy patient always wanting to control”* **FGD1K1**.

*“…Because I was nursing her to the best of my ability, she took advantage and called me for every little thing. Even things she could have done by herself*” **FGD1Y1**.

Generally, all students saw, the older adults as being forgetful and having gait issues.

*“………. they could not walk properly, sometimes we assist them to walk ……., their memory is affected. You can ask them if they have eaten and ………., they will say they haven’t eaten”* **FGD4, N**.

### Attitude

For this theme, students were asked to assess their attitude and that of registered nurses towards older adults. We enquired about the attitude of the students themselves towards older adults. Subthemes that emerged were the attitude of students towards older adults and the observed attitude of registered nurses towards care of older adults.

#### The attitude of students towards older adults

Participants assessed themselves in this theme and reported that they see older adult patients “ as their grandparents”(11), “ as patients (19)” and nursing them as “ an opportunity”(8), and “a duty”(22).

Eleven students reported that they saw the older adult patients as their grandparents.

*“I was really happy nursing the patient because I didn’t see her as a patient, but I took her as my grandmother. So, nursing her was like I’m just doing something for my relative”***FGD1, KO**.

There were also expressions by some participants that they saw nursing the older adults as an opportunity to learn because when their parents grow old, they would have to take care of them.

*“I see nursing them as an opportunity to learn as my parents will also grow old”* **FGD4, W**.

However, most students only take care of the older adults because they have been assigned to.

*“ Whenever I get to his bedside he frowns and puts up an attitude hmmm I could have equally given him an attitude … I decided to attend to him because it’s my duty”* **FGD2, KW2**.

#### The attitude of registered nurses

When asked to report on the attitude of staff nurses observed by students on the ward, the study reveals both the good and bad sides of some nurses. Participants reported seeing some nurses seen as kind and patient, humble and attending to calls of older patients.

*“I have also worked with some nurses who were kind to such patients.* **FGD4, B***“… they gave her*(the patient*) a bell so she can ring the bell to call for attention* (this is not normally practised in Ghana although it is in theory)*”* **FGD1, K**.

*“They were humble to the patients. … It was quite different from taking care of a younger person”* **FGD3, AK**.

Most of the participants (18), however, had observed negative attitudes exhibited by some nurses.

*“…others were rude because the patient was always complaining. We had to lift the patient whenever she wants to attend the washroom or do anything”* **FGD2, KW**.

All participants reported that nurses of higher ranks mostly attend to older adults who have been tagged “troublesome’.

*“The nurses on duty were running from attending to his daily care except for the In-charge nurse who took it upon herself to nurse him*” **FGD2, KW2**.

Additionally, almost all participants ascribed the neglect of the care of older adults to lower-ranked nurses.

*“…some of the nurses were not paying much attention to older adult patients. Rather sent us (students) to attend to the needs of such patients. Especially diaper changing and bathing. Because their issues are complicated, some nurses don’t want to get close*” **FGD4, J**.

### Career progression

We wanted to find the views of students on what is needed to care for older adults and whether they would be interested in a career progression as geriatric nurses. Subthemes were characteristics needed for career progression into geriatric nursing and desire to progress into geriatric nursing.

#### Career progression into geriatric nursing needs

Almost all the students were of the view that although in their training they have acquired skills and knowledge to take care of patients, for the older adults they need special skills and above all a lot of patience. Many admitted that they were not adequately prepared to properly nurse older adults yet.

*“We need more skills to be able to take care of them easily. We might have learnt a lot in our general nursing but if we can specialize or have more knowledge when caring for the aged…”* **FGD2, K2**.

*“As much as I believe nursing care is universal, when caring for the aged we need to do times 2 of what would have been done for an adolescent. Patience is a key characteristic needed*” **FGD4, A4**.

When we probed to identify how many students would like to work in the geriatric unit after graduation. After a 3 min think-through, 3 students managed to volunteer to say they would not mind but it was not going to be a first-choice option.

*“….well, I would not mind but that would be after I run out of options* (laughs)*”* **FGD2, K2**.

## Discussion

This study assessed the perceptions and attitudes of student nurses towards the care of older adults. The findings showed that students had a good perception of older adults and ageing. Most participants were of the view that when one is advanced in years, he/she is an older adult. The students also believed that being physically weak, frail not being able to handle much activity, as well as being forgetful identifies an older person. This is in agreement with studies by Ghimire, Shrestha, Callahan, Nath, Baral, Lekhak, and Singh [8]; Hakverdioğlu Yönt, Akin Korhan, and Dizer, [10]; Neville and Dickie [23]. This description within the study setting is not surprising as people retire at 60 years and get into a period of inactivity making them prone to various diseases. This makes most older adults degenerate faster than their counterparts in developed countries. Furthermore, some students opined that to be classified as an older adult is individualistic. Thus, it depends much on how one carries him or herself. This definition by a few students is in agreement with Ng and Indran [24] who advocate for a focus more on the role-centric approach when framing ageing and not chronological age.

The study concludes that the challenge was often with the reaction of the older adults towards students who took care of them. Some older adults did not understand why young nurses should be taking care of them so they either did not allow students near them or did not cooperate entirely with the students. This though unfortunate is what happens in our setting. Students in clinical practice are seen as “experimenters” so some patients would want the “experts” to attend to them. This sometimes limited the practice of the students as they are not allowed to nurse the older adults. Additionally, most students, who perceived older adults as boring, stated that they cared for them as part of their assigned duties but not their own will. This comment by students is also found in Bleijenberg [3], a study among Dutch undergraduate nurses.

According to the report of the participants, most nurses had negative attitudes towards older adults. However, it was not towards the person but the characteristics and complexities that accompany ageing. They reported issues of a diaper change and frequent complaints of the older adult. This confirms a review by Rush et al., ([30]) which suggested that the attitude of nurses were directed at the care demands of older adults.

This study found that somestudents had a positive attitude towards the older adults which confirms the study by Lee, Shin, & Greiner [18], that education can influence the attitude of students towards older adults. The level 400 students who formed the majority of participants, had been taught palliative care and introduction to gerontology in their final year. There is also a possibility that these students respected the older adults possibly because of the cultural background of the population. The composition of the study population and the results further contradicts a suggestion by McCloskey et al.,[21] which stated that placing the gerontological nursing course in the final year could increase interest in geriatrics. Most of the students had no increase in geriatric care. Similar to the study in Nigeria by Faronbi, Adebowale, Faronbi, Musa, & Ayamolowo [6], most of the students were not ready to work with geriatrics in the future. They saw geriatric care to be tedious, demanding, and needs a lot of patience which per their assessment they had not acquired. This result is similar to research by Natan, Danino, Freundlich, Barda, & Yosef [22], where students had no interest in working in geriatric units upon graduation. Majority of the students advocated for special training to enable nurses to take better care of the older adults just as was recommended by Abreu, & Caldevilla [1], Back et al., [2] and Özdemir, & Bilgili [26].

### Limitations and Strengths

Several limitations of this study that may influence our results must be addressed. First, due to the small size of this study and because the participants were limited to Ghanaian nursing students at a private university, the results are difficult to generalize to other populations. Also, although there are registered nurses in the regular program none volunteered to be part of the focus group discussions so views of the generic nurses may have been biased.

A strength of the study however is that being a qualitative study, the responses of the participants were a true reflection of what happens on the ground. Secondly, level 400 students who formed a majority of participants had been taken through the introduction to gerontology and palliative care course in the last semester so they had better knowledge on caring for older adults. To the best of our knowledge, no study has yet been conducted in Ghana looking into the perception and attitude of student nurses towards the care of older adults.

## Conclusions

Students generally had a positive perception about the older adults and accorded them the respect due them. They are fond of the older adult patients; however, they were not enthusiastic about nursing older adult patients. We recommend further in-depth training of nurses and student nurses on how to care for the older population possibly in separate units so that soon, our older adults will have an improved quality of life. Secondly, the better orientation of student nurses to older adult patients during clinical practice will also make older adult patients more receptive to them and enhance the young-old relationship.

### Implications for nursing education and practice

- Gerontology must be taught in more detail to make nursing students better equipped when they graduate to take care of older persons.
- Curriculum review in nursing may be necessary to introduce gerontology in the early years of training and also make the course more attractive.
- Most older patients do not cooperate with student nurses because they perceive student nurses as young and inexperienced. This is a challenge to rendering quality nursing care. Hence proper orientation of students to patients is very important.

## Data Availability

The datasets used and/or analysed during the current study are available from the corresponding author on reasonable request.

## Abbreviation

UN: United Nations
