# Correction to: *‘I decided to attend to him because it’s my duty’*: Student Nurses perception and attitude towards care of older adults

**DOI:** 10.1186/s12909-022-03129-9

**Published:** 2022-01-26

**Authors:** Priscilla Yeye Adumoah Attafuah, Ninon Amertil, Jacob Owusu Sarfo, David Atsu Deegbe, Delight Nyonator, Caleb Amponsah-Boama, Aaron A. Abuosi

**Affiliations:** 1 School of Nursing and Midwifery, University of Ghana, Legon, Ghana; 2 Department of Health Services Research and Care and Public Health Research Institute (CAPHRI), Maastricht University, Maastricht, the Netherlands; 3 School of Nursing and Midwifery, Valley View University, Oyibi, Ghana; 4 Department of Health, Physical Education and Recreation, University of Cape Coast, Cape Coast, Ghana; 5 Department of Public Admin and Health Services, University of Ghana Business School, Legon, Ghana

**Correction to: BMC Med Educ 22, 23 (2022)**

**https://doi.org/10.1186/s12909-021-03090-z**

Following publication of the original article [1], we have been informed that the authors were incorrectly affiliated.

The author affiliation has been updated above and the original article [1] has been corrected.

## References

[1] Attafuah (2022). *‘*I decided to attend to him because it’s my duty’: Student Nurses perception and attitude towards care of older adults. BMC Med Educ.

